# Prognostic value of PD-1, PD-L1 and PD-L2 deserves attention in head and neck cancer

**DOI:** 10.3389/fimmu.2022.988416

**Published:** 2022-09-02

**Authors:** Siqing Jiang, Xin Li, Lihua Huang, Zhensheng Xu, Jinguan Lin

**Affiliations:** ^1^ Department of Comprehensive Chemotherapy/Head and Neck Cancer, Hunan Cancer Hospital, The Affiliated Cancer Hospital of Xiangya School of Medicine, Central South University, Changsha, China; ^2^ Department of Pain Management and Anesthesiology, The Second Xiangya Hospital, Central South University, Changsha, China; ^3^ Center for Experimental Medicine, Third Xiangya Hospital of Central South University, Changsha, China; ^4^ Department of Oncologic Chemotheraphy, Affiliated Haikou Hospital of Xiangya Medical College, Central South University, Haikou, China

**Keywords:** PD-1, PD-L1, PD-L2, prognostic value, head and neck cancer, HNSCC, spatiotemporal heterogeneity

## Abstract

Head and neck cancer has high heterogeneity with poor prognosis, and emerging researches have been focusing on the prognostic markers of head and neck cancer. PD-L1 expression is an important basis for strategies of immunosuppressive treatment, but whether it has prognostic value is still controversial. Although meta-analysis on PD-L1 expression versus head and neck cancer prognosis has been performed, the conclusions are controversial. Since PD-L1 and PD-L2 are two receptors for PD-1, here we summarize and analyze the different prognostic values of PD-1, PD-L1, and PD-L2 in head and neck cancer in the context of different cell types, tissue localization and protein forms. We propose that for head and neck cancer, the risk warning value of PD-1/PD-L1 expression in precancerous lesions is worthy of attention, and the prognostic value of PD-L1 expression at different subcellular levels as well as the judgment convenience of prognostic value of PD-1, PD-L1, PD-L2 should be fully considered. The PD-L1 evaluation systems established based on immune checkpoint inhibitors (ICIs) are not fully suitable for the evaluation of PD-L1 prognosis in head and neck cancer. It is necessary to establish a new PD-L1 evaluation system based on the prognosis for further explorations. The prognostic value of PD-L1, PD-L2 expression in head and neck cancer may be different for early-stage and late-stage samples, and further stratification is required.

## 1 Introduction

Head and neck cancer (HNC) refers to tumors that occur in the lips, oral cavity, pharynx, larynx, and paranasal sinuses; occult primary cancers, salivary gland cancers and mucosal melanomas also deserve attention (1). About 90% head and neck cancers originating in the oral cavity, larynx, pharynx (hypopharynx, nasopharynx, or oropharynx) and sinus tract are squamous cell carcinoma (SCC) in pathological type (2, 3). There are approximately 880,000 new cases of head and neck squamous cell carcinoma (HNSCC) and more than 440,000 deaths worldwide annually (4). Therefore, prognostic judgement of HNC is helpful to disease prevention and treatment.

The PD-1/PD-L1 axis plays an important role in HNC therapy (5, 6). Among them, PD-L1 is not only a guidance for the use of immune checkpoint inhibitors (ICIs) (7), but also a potential prognostic indicator for head and neck cancer. Although studies on the prognosis of PD-L1 expression in head and neck cancer emerge one after another, the conclusions are controversial, and even the conclusions of some meta-analyses including large sample data are inconsistent (8–14). Meanwhile, PD-L2 is another important receptor of PD-1, and its binding affinity to PD-1 is about 2-6 times higher than that of PD-L1 (15). We have noticed a gradual increase in research on the prognostic value of PD-1 (CD279) and PD-L2 in HNC in recent years.

In terms of tumor development process, PD-1 and PD-L1 are also expressed in HNC precancerous lesions (16). From the perspective of subcellular localization of PD-L1 in tumor cells, there are membrane PD-L1, cytoplasmic PD-L1, and nuclear PD-L1 respectively (17). In terms of the distribution of tumor microenvironment at the cellular level, PD-1, PD-L1 and PD-L2 are not only expressed in tumor cells, but also in immune cells (18–21). In terms of protein forms, there are soluble PD-1 and PD-L1, and exosomal PD-L1, etc. (17). With the deepening of research, the prognostic value of PD-1, PD-L1 and PD-L2 indifferent localization and forms in head and neck cancer is not completely consistent. In the present review, we will comprehensively summarize the prognostic value of PD-1, PD-L1 and PD-L2 expression in HNC from different cellular localizations and forms mentioned above.

## 2 Precancerous lesions

In the precancerous lesions of laryngeal cancer, such as respiratory papilloma (22), actinic cheilitis (AC) (23), and oral leukoplakia (24), the expressions of both PD-1 and PD-L1 are up-regulated. There were differences in PD-1 and PD-L1 expression in the epithelium (E) and sub-epithelial (S) of oral lichen planus (OLP) with malignant transformation within 5 years, and increased PD-L1 levels were significantly associated with malignant transformation within 5 years. Studies have suggested that immune regulation through the PD-L1/PD-1 pathway occurs before the malignant transformation of oral precancerous lesions (16, 25). In a systematic review and meta-analysis of PD-L1 expression in head and neck precancerous lesions, PD-L1 appeared to be more frequently expressed in precancerous lesions than in normal mucosa, but to be less frequently expressed than in cancer lesions of invasive squamous cell carcinoma (26).

## 3 Subcellular level localization

The commonly used systems for detecting PD-L1 are 22C3, SP263, 28-8, SP142 and 73-10. Although these detection systems use different cut-off values ​​in the indications for ICI use (27), the staining targets are basically the same in both the cell membrane and endomembrane system of tumor cell (TC) and immune cell (IC) (28). PD-L1 positivity was defined as any partial or complete membrane staining for tumor cells, and both membrane and cytoplasmic staining for mononuclear inflammatory cells (lymphocytes and macrophages) (28). In both radioresistant (RR) and radiosensitive (RS) HNSCC cell lines, strong PD-L1 expression was found in the nuclear and cytoplasmic fractions of RR cancer cell lines, and PD-L1 was decreased in the nuclear fraction after irradiation but increased in the cytoplasmic fraction (29). This suggests that the nuclear localization of PD-L1 may reflect the disease prognosis by affecting the radiosensitivity.

## 4 Cell and tissue-level localization

### 4.1 Cell-level localization

In EBV-positive nasopharyngeal carcinoma (NPC), high PD-L1 expression on IC and TC is an independent favorable prognostic factor for overall survival (30). Also, PD-L1 expression in TCs is a favorable prognostic factor in NPC patients with pre-existing TILs (31). Among young patients with oral cavity squamous cell carcinoma (OCSCC), those with higher membrane PD-L1 positivity and the presence of TIL had a reduced risk of recurrence and improved survival (32).

#### 4.1.1 Tumor cells

In NPC, the patients with positive PD-1 staining on TC have a longer OS and progression-free survival (PFS), which is an independent prognostic factor for PFS (33). PD-1 mRNA over-expression in tumor tissue is associated with good prognosis in HNSCC patients treated with primary surgery, and low PD-1 mRNA levels are associated with the high risk of recurrence (34). In addition, in laryngeal squamous cell carcinoma (LSCC), the higher the expression of PD-1 in tumor tissue, the larger the tumor diameter (35).

In NPC patients, the uptake of 18F-FDG (18F-fluorodeoxyglucose) into NPC lesions was positively correlated with PD-L1 expression in TCs (36), the expression of PD-L1 in TC is positively correlated with T staging (37), and high PD-L1 expression is significantly associated with poor OS (38, 39) and DFS (40–42). The patients with concurrent chemoradiotherapy with high PD-L1 expression are more prone to complete response (CR) (43), allowing these patients to have a longer local-regional failure-free survival (LRFFS) time and slightly longer progression-free survival (PFS) (44). But it has also been suggested that increased PD-L1 expression is significantly associated with local failure after radiotherapy (45). Overall, PD-L1 over-expression in TCs of NPC is generally considered to be a poor prognostic factor. In oral squamous cell carcinoma (OSCC), PD-L1 expression in more than 10% of TCs was associated with tumor recurrence and lower disease-specific survival (46), and when the PD-L1 staining threshold was not considered, PD-L1 expression was positively correlated with the tumor size (47), lymph node metastasis at diagnosis, and overall tumor-related death (48). In the subgroup with oral submucous fibrosis (OSF), high PD-L1 expression resulted in a worse prognosis (49). Studies suggest that the prognostic significance of TC PD-L1 expression in oral cancer depends on the specificity of tumor site (50); PD-L1 upregulation in tongue squamous cell carcinoma (TSCC) is associated with a higher recurrence rate after tongue cancer surgery (51), later TNM staging and shorter PFS (52); but it was not correlated with OS (53). In SGCs, PD-L1 expression of TCs is positively correlated with tumor staging (54), and correlated with lymph node (LN) metastasis (55), postoperative recurrence and metastasis (56), and poor DFS (57). Compared with the PD-L1 low-expressing group, the OS of PD-L1 high-expressing cases was significantly shortened (56, 58), however, there was also opposite conclusion reported (59). In oral mucoepidermoid carcinomas (MECs), except that PD-L1 expression was positively correlated with histological grade, no relationship was observed between immunosuppressive proteins and other clinic pathological parameters (60). When PD-L1/PD-1 is co-expressed on TC, it can lead to NPC (33, 61), OSCC (62), TSCC (63), and hypopharyngeal carcinoma with poor prognosis (64), but there are still opposite observation (65).

PD-L2 is expressed in both the TC and stroma of HNSCC (66), and in a cohort of operable HNSCC patients, 62.7% of HNSCC tumors show positive staining of PD-L2, revealing PD-L2 an independent predictor of shorter OS (67). OSCC patients with higher PD-L2 score had a significantly worse prognosis (47, 68). PD-L2 over-expression is common in SGC, and it is associated with a decrease in disease-specific survival (DSS) and disease-free survival (DFS) (69), but there are also opposite conclusions reported (70).

#### 4.1.2 Immune cells

High percentage of PD-1-positivetumor-associated immune cells (TAICs) is an independent positive prognostic marker in oral OSCC (71). In oropharyngeal and hypopharyngeal carcinomas, Expression of PD-1 in tumor infiltrating lymphocyte (TIL) is significantly correlated with better overall survival (OS) and disease-free survival (DFS) (72). Nevertheless, in salivary gland carcinomas (SGC), conclusions of the prognosis judgment by the expression of PD-1 on TILs remains consistent (73, 74).

In NPC, the uptake of 18F-FDG (18F-fluorodeoxyglucose) into NPC lesions was negatively correlated with PD-L1 expression in tumor infiltrating immune cells (TIICs) (36), PD-L1 expression on TIL was negatively correlated with plasma EBV (Epstein-Barr virus) DNA load, N staging, M staging and clinical staging (37); It was also associated with significantly improved 1 and 2-year survival rates (75), and was positively correlated with the 5-year PFS rate (76). In EBV-positive NPCs, low PD-L1 expression in IC is an independent poor prognostic factor for DFS (30). However, in NPC patients with different ethnic backgrounds, PD-L1 expression in TILs had diametrically opposite effects on DFS (41, 42).

Further large-scale researches are needed to explore both the prognostic value of PD-L1 expression of TIL in OSCC (50, 77), and prognostic value of PD-L1 expression in tumor infiltrating mononuclear cells (TIMC) in salivary duct carcinoma (SDC) (56, 58). In TSCC, patients with high expression of PD-L1 in lymphocytes had better OS and RFS (78). PD-L1 expression in IC indicates better prognosis in LSCC and HPV-negative HNC (79).

### 4.2 Tissue-level localization

Approximately 19-46% of HNSCC patients have lymph node metastases at diagnosis (80, 81). In specimens of HNSCC primary cancer and corresponding lymph node metastases, PD-L1 expression in lymph node metastases was found to be significantly associated with decreased OS and DFS during oral chemotherapy treatment (72). With the development of separation technology of circulating tumor cells (CTCs) (82), the acquisition of CTCs has become more convenient. Although the expression of PD-L1 in tumor tissues is not completely consistent with the expression in CTCs (83), there are still studies suggesting that PD-L1 expression in CTCs has prognostic value. In a prospective study of locally advanced HNSCC patients treated with curative intent, the researchers have found that at the end of induction chemotherapy and concurrent chemotherapy, patients with PD-L1 mRNA over-expression in EpCAM(+) CTCs had shorter PFS and OS, so PD-L1 expression in EpCAM(+) CTCs is an independent prognostic factor for PFS and OS (84). In OSCC, high expression of PD-L1 in the CTC cytoplasm correlates with tumor size and LN metastasis and is an independent positive prognostic factor (85). Additionally, PD-L1 mRNA expression in peripheral blood may be an indicator of the presence of metastatic disease (N+) in OSCC (86) and has shown poor survival rate (87).

### 4.3 The prognostic value of PD-L1 evaluation systems

Currently, commonly used PD-L1 evaluation systems include tumor cell positive proportion score (i.e. tumor proportion score, TPS), combined positive score (CPS), and immune cell positive proportion score (IPS, IC score, etc.). Among the SGCs of unlimited pathological types, TPS and CPS were associated with histological grade, TPS-positive patients had poorer PFS and OS, while TPS was an independent prognostic factor (88); but CPS and IC scores had no effect on DFS or OS (54). In specific pathological types of SGC, IC score was associated with poor prognosis of PFS and OS (89). In LSCC, compared with CPS ≤ 1, CPS≥1 was correlated with lower postoperative recurrence rate (90) and longer DFS (91).

## 5 Different protein forms

### 5.1 Soluble protein

Soluble proteins are circulating proteins produced from alternative splicing of membrane proteins (92). In ICI-unresponsive patients, serum concentrations of soluble PD-L1 were significantly higher than ICI-responsive ones (93). In NPC, sPD-L1 expression is positively correlated with clinical staging (94), patients with high sPD-1 have a longer survival than those with low sPD-1 (95), but some scholars have suggested that sPD-1is not associated with prognosis (96). Soluble PD-1 and PD-L1 in peripheral blood of patients with recurrent/metastatic head and neck cell carcinoma (R/M HNSCC) did not affect prognosis (92). In OSCC, sPD-L1 expression correlates with clinical staging, tumor cell differentiation and lymph node status (97).

### 5.2 Exosomal proteins

With the rapid development of exosome and PD-L1 detection technologies (98), in animal models of HNSCC, exosomal PD-L1 levels may reflect the efficacy of antitumor drugs to a certain extent (99). In a phase I clinical trial of cetuximab, ipilimumab and radiation therapy, PD-L1(+) exosomes increased from baseline in patients with disease relapse (100). In a study by the International Union for Cancer Control (UICC), exosomal PD-L1 and COX-2 levels were higher in HNSCC stage III/IV patients than in stage I/II patients (101).

## 6 Combination indicators

The heterogeneity of HNC is strong, and the combination of PD-L1 with different localization and forms with other indicators helps to improve its strength as a prognostic indicator (102), as summarized in **Table 1**.

**Table 1:** Combination prognostic indicators for different HNCs.

Cancer type	Combination indicators	Reference
NPC	EBV DNA	(76) Hu B, Sun M, Wang Z, et al. Prognostic Value of Programmed Cell Death-Ligand 1 Expression in Tumor-Infiltrating Lymphocytes and Viral Load in Peripheral Blood Mononuclear Cells for Epstein-Barr Virus-Positive Nasopharyngeal Carcinoma[J]. Clin Chem, 2020,66(9):1219-1227.
	M2-like macrophages	(103) Deng R, Lu J, Liu X, et al. PD-L1 Expression is Highly Associated with Tumor-Associated Macrophage Infiltration in Nasopharyngeal Carcinoma[J]. Cancer Manag Res, 2020,12:11585-11596.
	CD8 + TIL	(104) Ono T, Azuma K, Kawahara A, et al. Prognostic stratification of patients with nasopharyngeal carcinoma based on tumor immune microenvironment[J]. Head Neck, 2018,40(9):2007-2019.
	CD3+TIL	(105) Al-Rajhi N, Soudy H, Ahmed S A, et al. CD3+T-lymphocyte infiltration is an independent prognostic factor for advanced nasopharyngeal carcinoma[J]. BMC Cancer, 2020,20(1):240.
	BRAF	(106) Cao Y, Chan K I, Xiao G, et al. Expression and clinical significance of PD-L1 and BRAF expression in nasopharyngeal carcinoma[J]. BMC Cancer, 2019,19(1):1022.
OSCC	Gender	(107) Wilms T, Gu X, Boldrup L, et al. PD-L1 in squamous cell carcinoma of the oral tongue shows gender-specific association with prognosis[J]. Oral Dis, 2020,26(7):1414-1423.
	Age	(32) Hanna GJ, Woo SB, Li YY, et al. Tumor PD-L1 expression is associated with improved survival and lower recurrence risk in young women with oral cavity squamous cell carcinoma. Int J Oral MaxillofacSurg, 2018,47(5):568-577.
	Smoking	(108) Lin Y M, Sung W W, Hsieh M J, et al. High PD-L1 Expression Correlates with Metastasis and Poor Prognosis in Oral Squamous Cell Carcinoma[J]. PLoS One, 2015,10(11):e142656.
	Metastatic lymph node necrosis	(109) Chen T C, Wu C T, Wang C P, et al. Associations among pretreatment tumor necrosis and the expression of HIF-1alpha and PD-L1 in advanced oral squamous cell carcinoma and the prognostic impact thereof[J]. Oral Oncol, 2015,51(11):1004-1010.
	CD8+TIL	(110) Kawaguchi T, Ono T, Sato F, et al. CD8+ T Cell Infiltration Predicts Chemoradiosensitivity in Nasopharyngeal or Oropharyngeal Cancer[J]. Laryngoscope, 2021,131(4):E1179-E1189.
	CD4+T cells	(111) Takahashi H, Sakakura K, Arisaka Y, et al. Clinical and Biological Significance of PD-L1 Expression Within the Tumor Microenvironment of Oral Squamous Cell Carcinoma[J]. Anticancer Res, 2019,39(6):3039-3046.
LSCC	TILs count	(112) Vassilakopoulou M, Avgeris M, Velcheti V, et al. Evaluation of PD-L1 Expression and Associated Tumor-Infiltrating Lymphocytes in Laryngeal Squamous Cell Carcinoma[J]. Clin Cancer Res, 2016,22(3):704-713. (113) Franz L, Alessandrini L, Fasanaro E, et al. Prognostic impact of neutrophils-to-lymphocytes ratio (NLR), PD-L1 expression, and tumor immune microenvironment in laryngeal cancer[J]. Ann Diagn Pathol, 2021,50:151657.
	CD8/FOXP3 TIL ratio	(114) Ono T, Azuma K, Kawahara A, et al. Predictive value of CD8/FOXP3 ratio combined with PD-L1 expression for radiosensitivity in patients with squamous cell carcinoma of the larynx receiving definitive radiation therapy[J]. Head Neck, 2020,42(12):3518-3530.
	HPV	(115) Yang S M, Wu M, Han F Y, et al. Role of HPV status and PD-L1 expression in prognosis of laryngeal squamous cell carcinoma[J]. Int J Clin Exp Pathol, 2021,14(1):107-115.
Hypopharyngeal carcinoma	CD8+ TIL	(116) Hu C, Tian S, Lin L, et al. Prognostic and clinicopathological significance of PD-L1 and tumor infiltrating lymphocytes in hypopharyngeal squamous cell carcinoma[J]. Oral Oncol, 2020,102:104560.
	CD4+TIL	(117) Shen L F, Zhou S H, Guo Y. Role of GLUT-1 in the Upregulation of PD-L1 Expression After Radiotherapy and Association of PD-L1 with Favourable Overall Survival in Hypopharyngeal Cancer[J]. Onco Targets Ther, 2020,13:11221-11235.
	FOXP3 and IL-10	(118) Sun J, Lian M, Ma H, et al. Competing endogenous RNA network analysis of CD274, IL10 and FOXP3 coexpression in laryngeal squamous cell carcinoma[J]. Mol Med Rep, 2018,17(3):3859-3869.
HNSCC	Persistent tumor-associated hypoxia	(119) Ruhle A, Grosu A L, Wiedenmann N, et al. Hypoxia dynamics on FMISO-PET in combination with PD-1/PD-L1 expression has an impact on the clinical outcome of patients with Head-and-neck Squamous Cell Carcinoma undergoing Chemoradiation[J]. Theranostics, 2020,10(20):9395-9406.
	MHC class I	(120) Yoo S H, Keam B, Ock C Y, et al. Prognostic value of the association between MHC class I downregulation and PD-L1 upregulation in head and neck squamous cell carcinoma patients[J]. Sci Rep, 2019,9(1):7680.
	P16 status	(121) Ou D, Adam J, Garberis I, et al. Clinical relevance of tumor infiltrating lymphocytes, PD-L1 expression and correlation with HPV/p16 in head and neck cancer treated with bio- or chemo-radiotherapy[J]. Oncoimmunology, 2017,6(9):e1341030.
	EMT	(122) Ock C Y, Kim S, Keam B, et al. PD-L1 expression is associated with epithelial-mesenchymal transition in head and neck squamous cell carcinoma[J]. Oncotarget, 2016,7(13):15901-15914.
	CD8 and CD4 T cell activation, natural killer cell and IFN activation	(123) Prat A, Navarro A, Pare L, et al. Immune-Related Gene Expression Profiling After PD-1 Blockade in Non-Small Cell Lung Carcinoma, Head and Neck Squamous Cell Carcinoma, and Melanoma[J]. Cancer Res, 2017, 77(13):3540-3550.
	IL-21 and Treg	(124) Zhao Y, Zhang Z, Lei W, et al. IL-21 Is an Accomplice of PD-L1 in the Induction of PD-1-Dependent Treg Generation in Head and Neck Cancer[J]. Front Oncol, 2021,11:648293.
	Radiosensitivity gene	(125) Dai D, Guo Y, Shui Y, et al. Combination of Radiosensitivity Gene Signature and PD-L1 Status Predicts Clinical Outcome of Patients with Locally Advanced Head and Neck Squamous Cell Carcinoma: A Study Based on The Cancer Genome Atlas Dataset[J]. Front Mol Biosci, 2021,8:775562.

## 7 Summary and outlook

**Figure 1:**
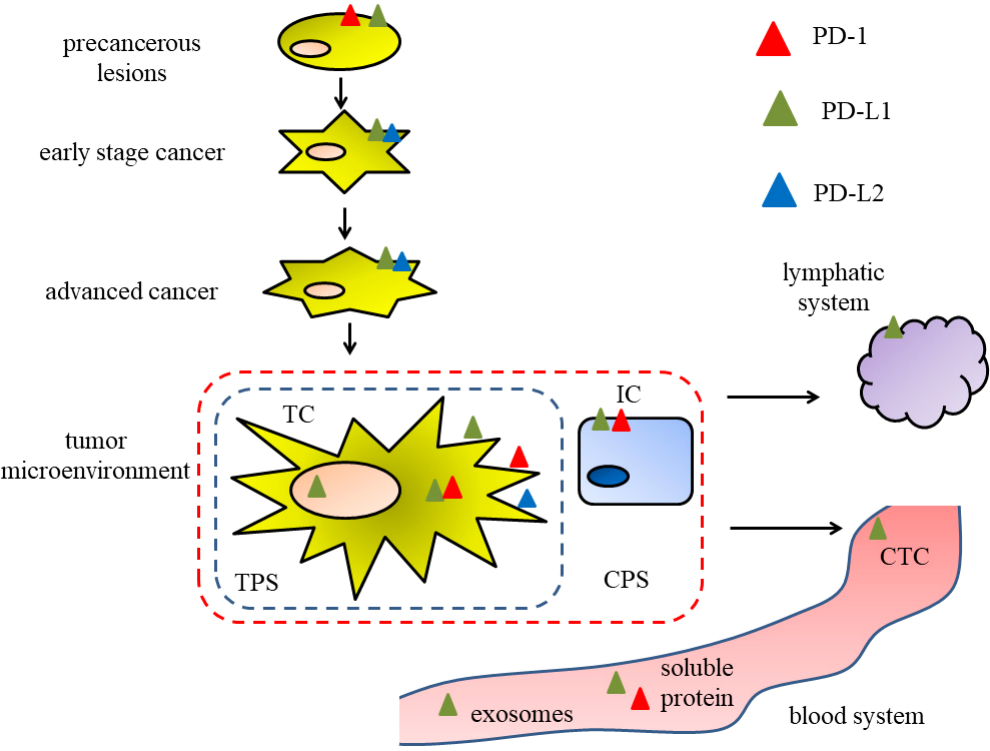
Figure 1 The different localizations and forms of PD-1, PD-L1 and PD-L2 in different stages of head and neck cancer, associating with the prognostic value.

In conclusion, it is of great significance to further expand the knowledge of the prognostic value of PD-1 and its receptors PD-L1 and PD-L2 in head and neck cancer. We summarize and analyze the different localizations and forms of PD-1, PD-L1 and PD-L2 with prognostic value in head and neck cancer, as shown in **Figure 1**. Interestingly, PD-1/PD-L1 expression in precancerous lesions may be a prognostic indicator for assessing the risk of malignant progression in precancerous lesions. Considering the effect of PD-L1 localization at different subcellular levels on radiosensitivity, the prognostic value of PD-L1 expression at the subcellular level deserves further exploration. At the same time, in terms of the convenience of judging the prognostic value of PD-1/PD-L1, it is necessary to expand the sample size to explore the possibility of prognostic value of PD-1/PD-L1/PD-L2 associated exosomes and related indicators. So far, the commonly used PD-L1evaluation systems (TPS, CPS, etc.) are based on the needs of ICI treatment. Considering the intratumor heterogeneity of PD-L1 expression (126), the sensitivity of the evaluation system (127, 128), the influence of factors such as specimens of different sources (27) on the expression of PD-L1, and that the expression pattern of specific immune checkpoint in HNSCC patients may also promote the development and efficacy of immunotherapy (129), all these urge us to pay attention to the importance of the expression pattern of PD-1/PD-L1 rather than to singly explore the expression level of PD-1/PD-L1. Some researchers reported that in OSCC, PD-L1 was expressed in a patchy or diffuse pattern in TCs by immunohistochemical method, and proposed that a mottled pattern was an independent risk factor for overall survival (130). Referring to this idea, if the prognostic value of PD-1/PD-L1 is to be more accurately described, new advanced prognostic evaluation criteria for PD-1/PD-L1 will be established.

We also note that although PD-L1 expression on TCs is an unfavorable prognostic factor in OSCC, in locally advanced oral squamous cell carcinoma (LAOSCC), positive PD-L1 expression is significantly associated with higher disease-free survival and overall survival in patients (131). In operable HNSCC patients, PD-L1 positivity in TC was an independent factor for poor PFS in young patients (<45 years) (132), and is also an independent risk factor for OS and DFS (133, 134). However, in R/M HNSCC treated with standard-of-care chemotherapy, PD-L1 expression in TC was not considered to predict the patient’s OS (135). Similarly, although PD-L2 expression in TC was an independent prognostic factor for poor OS in operable HNSCC, in a small clinical study of R/M HNSCC, the PFS and the median time for overall survival in patients with positive PD-L2 expression were significantly longer than PD-L2-negative patients (20). The difference between the above conclusions suggests that the prognostic value of specimens obtained at different disease stages is different even if the same indicators are detected.

In conclusion, PD-1, PD-L1 and PD-L2 may have different prognostic values in head and neck cancer considering their expression in different cell types, their tissue localization and their different protein forms. The clinical data, association and mechanisms of PD-1, PD-L1 and PD-L2 prognostic values in head and neck cancer deserve further studies.

## Author contributions

SJ wrote the article, XL provided drawing support, LH provided language editing assistance, ZX revised the article, and JL provided the publication fee. All authors contributed to the article and approved the submitted version.

## Conflict of interest

The authors declare that the research was conducted in the absence of any commercial or financial relationships that could be construed as a potential conflict of interest.

## Publisher’s note

All claims expressed in this article are solely those of the authors and do not necessarily represent those of their affiliated organizations, or those of the publisher, the editors and the reviewers. Any product that may be evaluated in this article, or claim that may be made by its manufacturer, is not guaranteed or endorsed by the publisher.
